# Bois noir management in vineyard: a review on effective and promising control strategies

**DOI:** 10.3389/fpls.2024.1364241

**Published:** 2024-03-27

**Authors:** Roberto Pierro, Abdelhameed Moussa, Nicola Mori, Carmine Marcone, Fabio Quaglino, Gianfranco Romanazzi

**Affiliations:** ^1^ Department of Pharmacy, University of Salerno, Fisciano, Salerno, Italy; ^2^ Pests and Plant Protection Department, Agricultural & Biological Research Institute, National Research Centre, Cairo, Egypt; ^3^ Department of Biotechnology, University of Verona, Verona, Italy; ^4^ Department of Agricultural and Environmental Sciences – Production, Landscape, Agroenergy, University of Milan, Milan, Italy; ^5^ Department of Agricultural, Food and Environmental Sciences, Marche Polytechnic University, Ancona, Italy

**Keywords:** *'Candidatus* Phytoplasma solani’, grapevine yellows, insect vectors, phytoplasma diseases, sustainable management strategies

## Abstract

Among grapevine yellows, Bois noir (BN), associated with ‘*Candidatus* Phytoplasma solani’, represents the biggest threat in the main wine-growing areas worldwide, causing significant losses in berry quality and yields. BN epidemiology involves multiple plant hosts and several insect vectors, making considerably complex the development of effective management strategies. Since application of insecticides on the grapevine canopy is not effective to manage vectors, BN management includes an integrated approach based on treatments to the canopy to make the plant more resistant to the pathogen and/or inhibit the vector feeding, and actions on reservoir plants to reduce possibilities that the vector reaches the grapevine and transmit the phytoplasma. Innovative sustainable strategies developed in the last twenty years to improve the BN management are reviewed and discussed.

## Introduction

Bois noir (BN) is the most important disease of the phytoplasma-associated grapevine yellows (GY) complex, causing important economic losses in all major wine-growing areas by reducing fruit quality and yields (Belli et al., 2010; Maixner, 2011). BN has been largely reported in the Euro-Mediterranean basin, South America, Asia, and Middle East (Bertaccini et al., 2014; Pierro et al., 2019).

**Figure 1:**
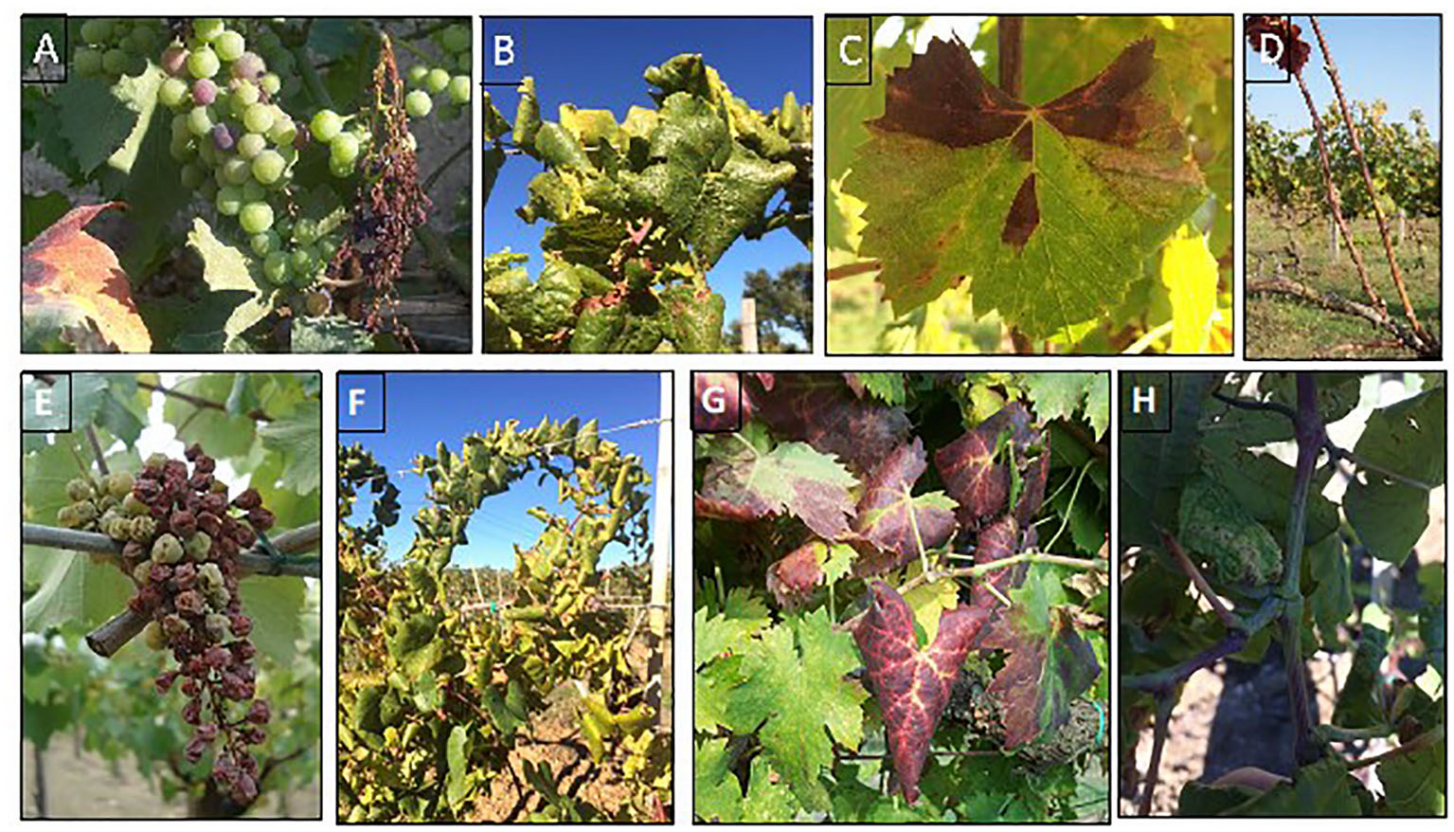
Typical symptoms associated with *‘Candidatus* Phytoplasma solani’ in *Vitis vinifera*: berry shriveling **(A, E)**, leaf yellowing and curling in white cultivars **(B, F)**, leaf reddening and curling in red cultivars **(C, G)**, incomplete shoot lignification **(D, H)**.

In grapevine (*Vitis vinifera* L.), BN showed typical GY symptoms, including leaf yellowing, or reddening (in white and red cultivars, respectively), downward leaf curling, flower abortion, berry shriveling , irregular ripening, and plant decline (**Figure 1**). In some cases, death can occur in 2 or 3 years from the infection (Belli et al., 2010). Nevertheless, symptom expression and intensity might be partially influenced by grapevine cultivars, rootstock, and environmental conditions (Romanazzi and Murolo, 2008; Maixner, 2011; Romanazzi et al., 2013).

**Figure 2:**
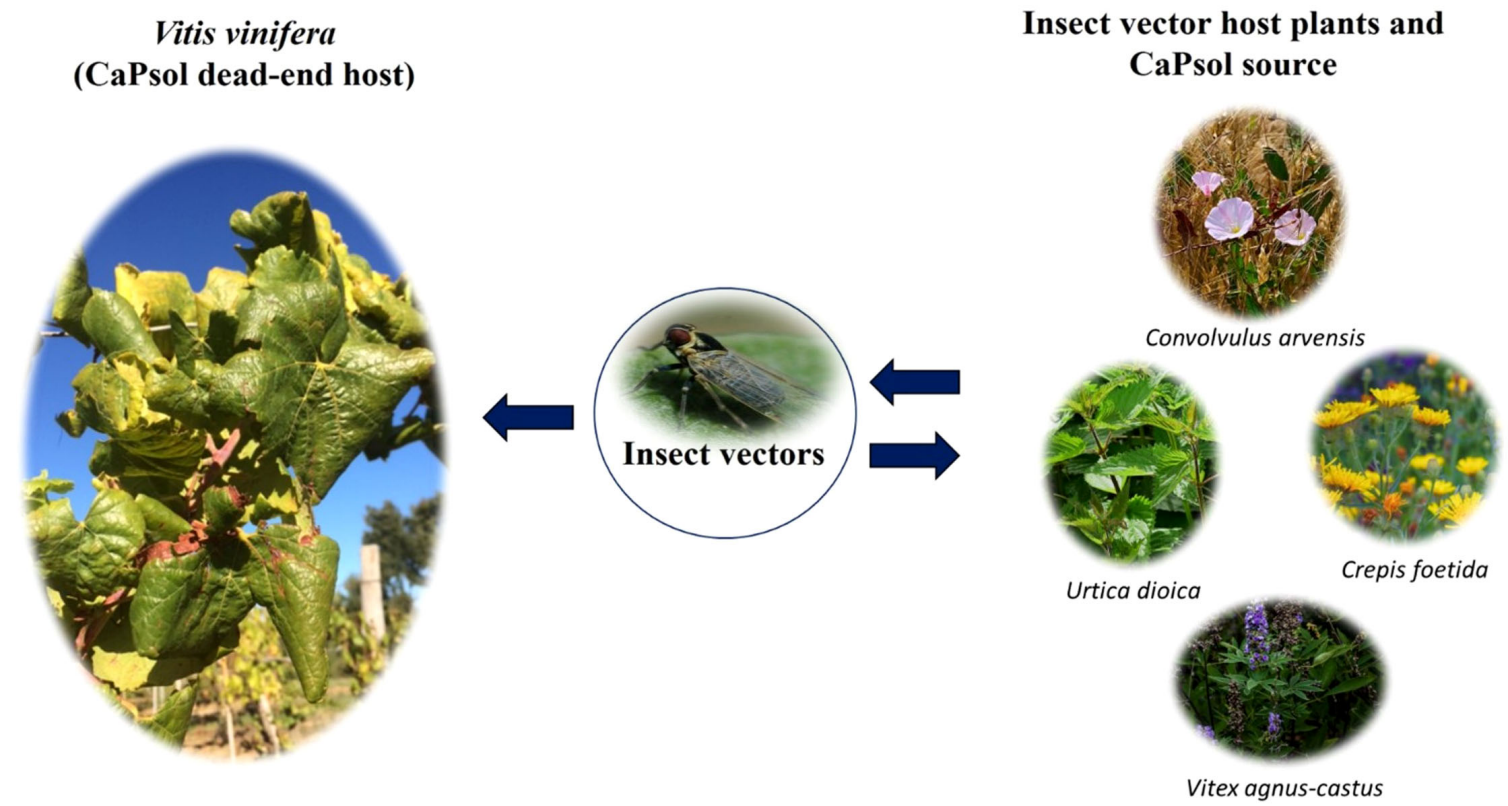
Epidemiological cycle of *‘Candidatus* Phytoplasma solani’: phytoplasma transmission is mainly vectored by *Hyalesthes obsoletus* from inoculum sources, such as *Urtica dioica, Crepis foetida, Vitex agnus-castus* and *Convolvulus arvensis*, to grapevine, representing a dead-end host.

BN has been associated with ‘*Candidatus* Phytoplasma solani’ (CaPsol, subgroup 16SrXII-A or stolbur group) (Quaglino et al., 2013). In Europe, Middle East, and Asia, the he main insect vector of CaPsol is the polyphagous planthopper *Hyalesthes obsoletus* Signoret (Maixner, 1994), erratically transmitting CaPsol to grapevine (Lessio et al., 2007) and living preferentially on stinging nettle (*Urtica dioica* L.) and field bindweed (*Convolvulus arvensis* L.), and also on mugwort (*Artemisia vulgaris* L.), stinking hawk’s-beard (*Crepis foetida* L.), lavender (*Lavandula spica* L.), chaste tree (*Vitex agnus-castus* L.), and muscat sage (*Salvia sclarea* L.) (Kosovac et al., 2016; Chuche et al., 2018; Mori et al., 2020; Sforza et al., 1999) (**Figure 2**). In Chile, CaPsol was found in Grapevine (Gajardo et al., 2009), but no information on vectors is reported.

In the last decades, multilocus typing sequence analyses were carried out to understand the genetic diversity of CaPsol in different geographic locations and gain information concerning its ecological niches and epidemiological patterns. Analyses on housekeeping and membrane protein genes were successfully adopted to comprehend the multiple ways of spreading of genetically different CaPsol strains and their mechanisms of interactions with host(s) (Langer and Maixner, 2004; Cimerman et al., 2009; Fialova et al., 2009; Pacifico et al., 2009; Lee et al., 2010; Murolo et al., 2010). Molecular approaches along with transmission trials unveiled that other insect species can play a role in vectoring CaPsol to grapevine, including *Reptalus panzeri* Löw*, Aphrodes makarovi* Zachvatkin*, Dicranotropis remaniaca* Guglielmino, D’Urso & Bückle*, Dictyophara europaea* (L.)*, Euscelis incisus* (Kirschbaum)*, Euscelidius variegatus* (Kirschbaum)*, Laodelphax striatella* (Fallén)*, Philaenus spumarius* (L.), and *Psammotettix* alienus and *P.* confinis (Dahbom) (Jović et al., 2009; Kosovac et al., 2016; Quaglino et al., 2019). In addition, *Reptalus artemisiae* (Becker)*, Macrosteles quadripunctulatus* (Kirschbaum)*, Anaceratagallia ribauti* (Ossiannilsson), and *Pentastiridius leporinus* (L.) were also identified as putative CaPsol insect vectors, still lacking defined epidemiological role (Riedle-Bauer et al., 2008; Maixner, 2011; Pierro et al., 2020). Moreover, many other weedy hosts were found infected by CaPsol, showing the existence of several reservoir plants in the different agroecosystems (Mori et al., 2015; Chuche et al., 2018; Quaglino et al., 2021; Moussa et al., 2023).

On the one hand, the multifaceted ecology of BN (**Figure 2**), including different vector populations, several herbaceous hosts and grapevine, highlighted the great genetic diversity and adaptability of phytoplasma, on the other hand, epidemiological complexity made difficult the development of effective management strategies against the disease (Maixner, 2011; Bertaccini et al., 2014).

It is known that tetracycline exhibited antibiotic activity against a wide range of microorganisms, including phytoplasma (Bertaccini et al., 2014), but use of antibiotics is not allowed in agriculture in the majority of the Countries/Continents (e.g., Europe, USA) to avoid insurgence of resistance in the pathogen(s) and hazard for human health. BN monitoring programs represent one of the most important steps for the identification of the phytoplasma disease in a specific geographic macro area. In the last decades, new approaches including the use of rapid loop-mediated isothermal amplification (LAMP) assays, vision-based systems by using artificial intelligence and drones significantly improved the detection of grapevine yellows (Kogovšek et al., 2017; Cruz et al., 2019; Ampatzidis et al., 2020; Matić et al., 2022). In vineyard, management strategies so far adopted for limiting phytoplasma outbreaks are based on: (i) use of phytoplasma-free propagating material and eradication of infected plants, (ii) vectors control, using insecticides on grapevine canopy, herbicides on ground cover to kill the insect’s wild host plants, or other agronomic practices. Novel, effective, and sustainable management strategies against phytoplasma diseases are developing worldwide, and the promising advances in the cultivation of phytoplasma (Contaldo et al., 2013) could support advances in the identification of effective compounds.

The aim of this review is to summarize the sustainable strategies developed in the last twenty years to improve BN management. Their effectiveness in contrasting disease spreading and obtaining recovered plants are reported, as well as the possible implications linked to specific geographical areas and environmental conditions.

## Bois noir management strategies focused on grapevine

**Figure 3:**
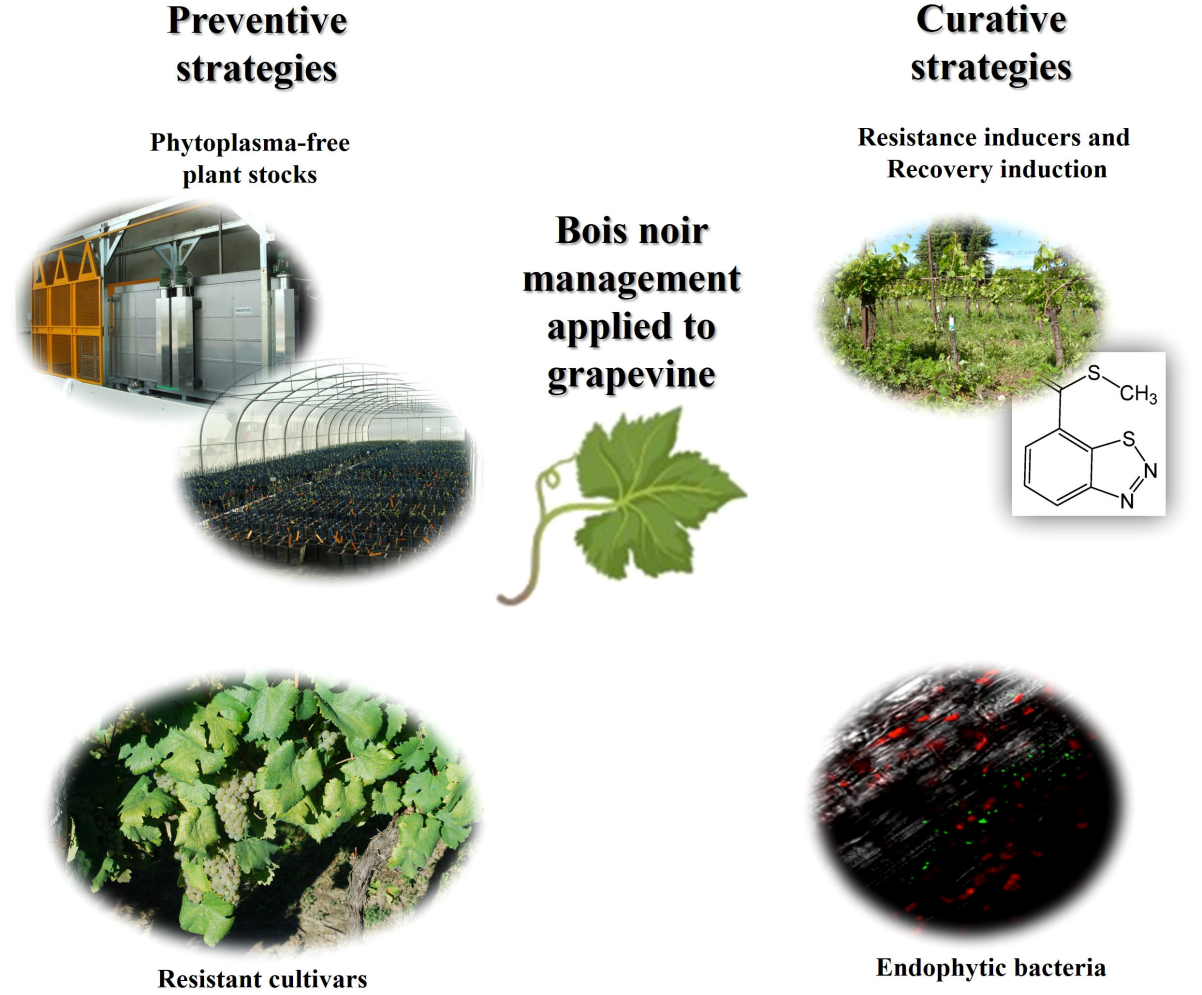
Sustainable Bois noir control strategies applied on grapevines.

**Figure 3** summarizes the Bois noir management strategies focused on grapevine.

### Utilization of lowly susceptible grapevine cultivars

The selection of healthy propagating material and the choice of grapevine varieties showing low susceptibility to the pathogen, can play a key role in the prevention of BN (Borgo and Angelini, 2002; Bianco et al., 2011; Malembic-Maher et al., 2020).

One of the most stimulating challenges would be represented by the identification and the selection of defense genes to transfer into new grapevine varieties, within a plant breeding program (Bianco et al., 2011).

Previous studies conducted on phytoplasma strains associated with BN, Flavescence dorée, elm yellows and apple proliferation, have shown some differences in symptom severity observed in field, depending on: (i) the different susceptibility level of plant varieties (Sinclair et al., 2000; Quaglino et al., 2016; Casarin et al., 2023), (ii) phytoplasma abundance in the host (Griffiths et al., 1999; Martini et al., 2011), (iii) genetic diversity of non-ribosomal phytoplasma genetic traits (Hren et al., 2009; Seemüller et al., 2013; Murolo and Romanazzi, 2015), and (iv) climatic conditions (Maixner, 2011; Romanazzi et al., 2013).

A wide difference in susceptibility of grapevine cultivars to BN is reported (Borgo and Angelini, 2002; Panassiti et al., 2015; Quaglino et al., 2016; Pierro et al., 2018), and ‘Chardonnay’ is known as one of the most susceptible varieties since it is rare to find a vineyard with such cultivar free of BN (Murolo et al., 2010), and the number of symptomatic plants can also reach the 60% (Murolo et al., 2020). The disease has an evolution over time, starting with a high progression to a maximum peak, followed by a regression phase related to the spontaneous remission of BN symptoms (Osler et al., 1993; Maixner, 2006; Murolo et al., 2020). Chardonnay infected by BN showed a reduced reactivity to BN as compared to the moderately susceptible Sangiovese (Landi and Romanazzi, 2011).

The Georgian *V. vinifera* germplasm has distinguished itself for cultivars characterized by specific response to abiotic stress and resistance traits related to biotic stress, including *Plasmopara viticola* and phytoplasma (Sargolzaei et al., 2021). A study conducted on vineyards of Khaketi and Shida Kartli regions, in eastern Georgia, have reported the presence of lowly susceptible cultivars, with mild GY symptoms and unaltered berry production in most of autochthonous grapevine varieties (Quaglino et al., 2016). Interestingly, the lowest GY susceptibility was observed in the Georgian local cultivars infected by phytoplasma strains causing severe symptoms on the international cultivars, unveiling the existence of specific genomic traits involved in the pathogenicity process and symptom patterns, which should be deeper investigated. Similarly, a study conducted in Sangiovese vineyards (Central Italy) have shown that CaPsol strains grouping in different phylogenetic clusters can cause different symptom intensity within the same grapevine cultivars and climatic conditions (Pierro et al., 2018; Pierro et al., 2018a), highlighting that genetically different phytoplasma strains have distinct virulence range.

Moreover, surveys conducted in different *Prunus* species affected by ‘*Candidatus* Phytoplasma prunorum’ showed that phytoplasma concentration in plant hosts can affect symptom expression (Martini et al., 2011). Although this correlation should be confirmed in grapevines affected by CaPsol strains, recovered plants have shown a phytoplasma titer in leaf veins and roots much lower than symptomatic plants (Landi et al., 2019).

Future challenges in this field are focused on the identification of grapevine genetic traits involved in the regulation mechanisms capable of conferring low susceptibility to BN , within a breeding program aimed at obtaining novel grapevine cultivars by horizontal gene transfer.

### Phytoplasma elimination from plant material

Plant diseases and their spreading can significantly decrease using healthy planting materials. Around half of the 19^th^ century, Morel and Martin (1952) published a new methodology to obtain virus-free plants from virus-infected plants using the meristem culture. This novel technique opened a new research field aimed at obtaining healthy propagating materials of several plant species affected by different pathogens, including phytoplasma. Up to now, phytoplasma-free plant stocks have been obtained through: (i) shoot tip culture (Chalak et al., 2005), (ii) thermotherapy (Křizan et al., 2008), (iii) embryogenesis (Parmessur et al., 2002), (iv) organogenesis (Wei et al., 2013), (v) stem culture (Dai et al., 1997), (vi) micrografting (Chalak et al., 2005), (vii) treatment of plant tissues with antibiotics (Chalak et al., 2005; Bertaccini, 2021), and (viii) cryotherapy of shoot tips (Wang and Valkonen, 2008), (ix) inducing recovery by using agronomical practices, such as uprooting, pruning or use of resistance inducers (see the next paragraph).

Thermotherapy of propagation material by hot water or hot air, combined with shoot tip culture, were largely adopted for phytoplasma elimination in many plant species, showing good sanitation rate (Bertaccini, 2021). In grapevine, hot water treatments, cryotherapy, and tissue cultures allowed the elimination of phytoplasma from infected mother plants (Caudwell et al., 1997; Gribaudo et al., 2007; Křizan et al., 2008; Wang and Valkonen, 2008), underlying the possible and promising applications of such methodologies on a large scale. Further research is needed to know the suspected influence of heat treatment on endophytic mycoflora.

Use of these techniques implies the checking of the effective elimination of phytoplasma in the treated plant materials. Nevertheless, after the introduction of phytoplasma-free planting materials in vineyards, the presence of insect vectors able to transfer phytoplasma into healthy plants still represents a concrete risk of new outbreaks. Thus, the BN management strategy needs to be implemented by multiple management approaches.

### Recovery induction in grapevine by agronomical practices and resistance inducers

The spontaneous remission of symptoms along with the elimination of the pathogen from the canopy, known also as recovery, can occur in symptomatic grapevines affected by phytoplasma diseases (Osler et al., 1993). Recovery represents a phenomenon not completely understood, hiding interesting aspects concerning the complex plant-pathogen interactions that could be utilized for the development of effective strategies for BN management. Recently, it was demonstrated that the expression of salicylate signaling pathway genes is activated in grapevines showing symptoms after CaPsol infection, antagonizing the expression of jasmonate signaling pathway genes. On the other hand, the jasmonate signaling pathway genes are over-expressed in BN-recovered plants, indicating the potential significance of jasmonate-regulated defenses in preventing CaPsol infections and the consequent BN onset. Consequently, recovery can be improved and maintained by inhibiting the activation of defense genes linked to salicylate signaling, while concurrently triggering jasmonate signaling and other defense mechanisms (Paolacci et al., 2017). Several studies reported that recovery can be induced by agronomical practices, such as grapevine partial uprooting and pulling (Osler et al., 1993; Maixner, 2006; Romanazzi and Murolo, 2008), pruning or pollarding of the symptomatic portions (Credi et al., 2008; Riedle-Bauer et al., 2010) and by applications of resistance inducers (Garau et al., 2007; Romanazzi et al., 2013; Moussa et al., 2021).

Concerning the agronomical practices, in the Marche region (Central Italy), partial uprooting of BN symptomatic grapevines induced recovery in the majority of cultivar Chardonnay, Verdicchio and Sangiovese grafted onto Kober 5BB, while a lower effectiveness was observed in the same cultivars grafted on 420A, unveiling an effect of the rootstock (Osler et al., 2003; Romanazzi and Murolo, 2008). Alternative agronomical practices, strongly influencing recovery of BN-infected vines and reducing its spreading within the vineyard, is represented by heavy winter pruning or pollarding of the symptomatic portions (Riedle-Bauer et al., 2010). The extensive removal of infected woods during the winter significantly improved the phenomenon of symptom remission yearly and in the next growing seasons, with variable recovery rates depending on grapevine cultivar, plant age, hardness of pruning, rootstock, and symptom intensity (Credi et al., 2008; Ipach et al., 2008; Stark-Urnau and Kast, 2008; Riedle-Bauer et al., 2010). Trials run by Osler and coworkers (2003) showed a very low number of symptomatic plants (1-2%) obtained grafting symptomatic materials on healthy rootstock. The possibility to elicit symptom remission can be also represented by grafting of recovered material on symptomatic grapevines. In field trials conducted in Franciacorta (Northern Italy), top-grafting of recovered shoots in cultivar Chardonnay showed curative effects on symptomatic plants, inducing recovery rates four times higher than in non top-grafted plants (Moussa et al., 2022). Moreover, the adoption of this technique allows a lower subsequent symptom development in recovered plants, reinforcing the effectiveness of such curative strategy in a sustainable BN management.

An important method to enhance defense responses of plants against pathogens reducing symptom appearance is the spray application of resistance inducers, known also as elicitors . Resistance inducers can activate stress-related defense pathways in the plants and modify the plant-pathogen-vector interactions affecting the emission of plant volatile compounds able to attract or repel insect vectors (Dicke and Hilker, 2003; Minuz et al., 2020).

In the case of BN, promising control strategies are represented by resistance inducers sprayed on canopy, that were able to increase the natural recovery rate in naturally CaPsol-infected grapevines (curative action) and/or limiting the transmission of CaPsol to healthy vines, interfering with the plant-vector relationship (preventive action).

First field applications of resistance inducers on grapevine were conducted in Sardinia on CaPsol-symptomatic plants of the cultivar Chardonnay, using formulations based on benzothiadiazole or glutathione in different concentrations (Garau et al., 2007). In this study, intensive applications (10-12 weekly treatments from mid-May to end of July) on grapevines achieved a statistically significant yield increase in treated plants in comparison with the untreated ones .

Similar studies were conducted in cultivar Chardonnay vineyards located in the Marche region, evaluating the effectiveness of 5 compounds (chitosan, phosetyl-Al, 2 formulations of glutathione plus oligosaccharines, and benzothiadiazole) as resistance inducers impacting on the symptom remissions and berry production, once sprayed weekly on canopy from the beginning of May to the end of July (Romanazzi et al., 2013). Benzothiadiazole miming the action of the salicylic acid, halved the number of symptomatic plants in the examined vineyards over a four- year trial . Effective recovery induction was also obtained using the formulations of glutathione plus oligosaccharines, likely for its protective role in cellular metabolism, removal of free radicals and transport of reduced sulfur. An induction of resistance was also observed on treated plants that retained disease symptoms, since the vines sprayed with the most effective compounds showed a reduced symptom severity, and over 80% of recovered plants did not show any more symptoms in the following years (Romanazzi et al., 2013).

In the last years, commercial products based on animal amino acids, seaweed (*Ascophyllum nodosum*) extract and plant-derived amino acids, which use as biostimulants is allowed in organic farming, were evaluated as elicitors for their curative and preventive actions in BN management in northern Italy. Based on the obtained results, it emerged that a reduction in the percentage of symptomatic vines was observed exclusively in the vineyards treated with animal amino acids, mainly for its curative effect (induction of recovery) (Moussa et al., 2021). Open field trials in different grape growing areas and on different cultivars are in progress to prove the correlation between biostimulant treatment and recovery induction, and confirm the effectiveness of biostimulants in BN control.

In 2008, studies based on the evaluation of resistance inducers were carried out in laboratory conditions. In the trials run by Bressan et al. (2008) is not completely clear if the effects of benzothiadiazole is just on the plant, just on vector or both, since this active ingredient is reported to have a wide eliciting activity, together with a less known antimicrobial effects toward pests and pathogens (Bressan et al., 2008; Romanazzi et al., 2013). Studies evaluating the responses of the phloem-feeding planthopper *H. obsoletus* to volatiles emitted by resistance inducer-treated grapevines unveiled that insect specimens were significantly repelled by volatile compounds emitted 7 days after the treatments of grapevine shoots with benzothiadiazole (Minuz et al., 2020). Based on this evidence and those previously reported, we can say that benzothiadiazole is involved in the plant systemic acquired resistance (SAR) mechanisms and it can influence planthopper behavior, thanks to changes in the volatile compound emissions, showing its potential key role in the sustainable management strategies for the control of BN. Regarding the side effect, benzothiadiazole showed “No bee precaution” (Bee Precaution Pesticide Ratings) and harmless on auxiliary fauna (IOBC-WPRS Pesticide Side Effect Database).

In conclusion, the utilization of agronomical practices (partial uprooting and pulling of symptomatic plants, grafting of materials from recovered vines to symptomatic plants) and the treatments of the canopy of BN-affected grapevines with resistance inducers or biostimulants have shown promising results in inducing recovery and increasing berry production in some grapevine cultivars. The application of these strategies should be extended to wider geographic areas and grapevine varieties to have further confirmation of their effectiveness, optimizing the formulations of resistance inducers, and clarifying the involved mechanism of actions.

### Employment of non-pathogenic endophytic bacteria in grapevine

Biological control is part of sustainable methods aimed at inhibiting plant pathogens through the competition for space and nutrients, improving plant immunity and/or obtaining beneficial effects from compounds released by specific microorganisms. It was demonstrated that non-pathogenic endophytic bacteria can contribute to plant growth and health, alleviation of plant stress, and in-planta contaminant-degradation (Yang et al., 2019), opening novel avenues for experimental activities focused on the identification of possible bacteria acting as biocontrol agents towards phytoplasma.

Previous evidence has reported relevant differences in endophyte populations between CaPsol infected vs. healthy grapevines, suggesting that phytoplasma can shape the bacterial community by selecting endophytic strains that could elicit a plant defense response (Bulgari et al., 2011). Investigations on endophytic communities in plants have stimulated further studies aimed at determining the efficient way to introduce specific endophyte species into mature vines under field conditions to reduce symptom severity of yellows disease.

In this challenging scenario, the Gram-negative endophytic bacterium *Frateuria defendens* (formerly referred to *Dyella*-like bacterium), isolated from the guts of *H. obsoletus*, was successfully introduced systemically into several plant genera (*Cucumis, Gossypium, Capsicum, Nicotiana, Catharanthus, Vitex*, and *Sesamum*), showing a significant reduction of phytoplasma titer and symptom intensity in plant (Naor et al., 2011; Iasur-Kruh et al., 2017; Lidor et al., 2018; Naama-Amar et al., 2020). Since phytoplasma cannot be grown in axenic culture, efficacy of *F. defendens* was firstly tested in periwinkles artificially infected by different phytoplasmas that cause disease in multiple host plants (Hodgetts et al., 2013). In 2018, *F. defendens* was introduced into phytoplasma-infected Chardonnay plantlets, strongly reducing symptom intensity (Iasur-Kruh et al., 2018; Naor et al., 2019; Naama-Amar et al., 2020). Two main hypotheses were proposed to explain antibiosis activities of *F. defendens*: the colonization of the same ecological niche (phloem) and the ability to limit and/or inhibit physiological activities of the pathogen (Iasur-Kruh et al., 2018), or the secretion of specific antimicrobial metabolites (Naama-Amar et al., 2020).

Spraying was found as the best method for an efficient penetration of *F. defendens* in the plant tissues. Moreover, the capability of the biocontrol agent to move along grapevine shoots also in non-treated plant parts increases its potential as a biocontrol agent (Iasur-Kruh et al., 2018; Lidor et al., 2018).

These promising results represent an excellent starting point for wider studies aimed at evaluating the effectiveness of *F. defendens* as a biocontrol agent in further grapevine cultivars and different environmental conditions.

## Bois noir management strategies focused on insect vectors

**Figure 4:**
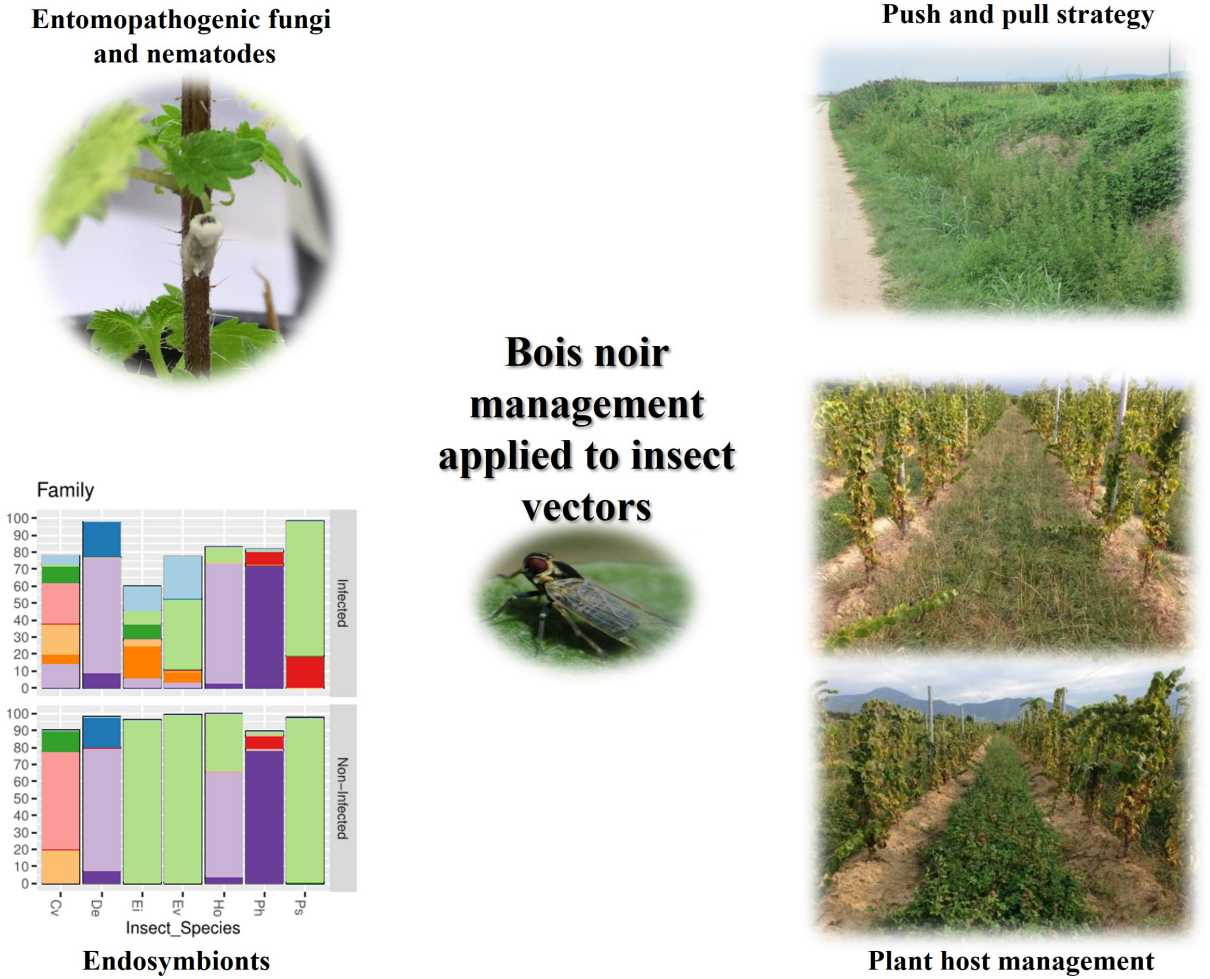
Sustainable Bois noir control strategies applied on insect vectors.

**Figure 4** summarizes the Bois noir management strategies focused on insect vectors.

### Application of entomopathogenic fungi and nematodes as *Hyalesthes obsoletus* biocontrol agents

Numerous studies reported that entomopathogenic fungi (EPFs) and nematodes (EPNs) can affect insects. An effective and novel integrated management against phytoplasma-associated diseases should include the identification and the control of phytoplasma insect vectors using biocontrol agents, such as EPNs and EPFs. In laboratory condition, Guerrero and Pardey (2019) showed the efficacy of EPNs belonging to the genus *Steinernema* and *Heterorhabditis* in controlling nymphs of *Haplaxius crudus* (Van Duzee), known to be the insect vector of ‘*Candidatus* Phytoplasma palmae’.

Similar experimental trials aimed at reducing *H. obsoletus* population using EPFs and EPNs were conducted under laboratory and greenhouse conditions. Results showed a high *H. obsoletus* adult and nymph mortality when the nematodes *Steinernema carpocapsae* (Weiser), *S. feltiae* (Filipjev), *Heterorhabditis bacteriophora* Poinar and the fungi *Metarhizium anisopliae* (Metschn.), *Cordyceps fumosorosea* (Wize), *Akanthomyces muscarius* (Petch) were applied (Langer et al., 2005; Moussa et al., 2021a). However, field trials highlighted that environmental conditions, such as soil moisture and salinity, can affect the EPFs and EPNs activity (Guerrero and Pardey, 2019); therefore, further studies to investigate their effectiveness under different climatic and environmental parameters, are necessary. Additionally, the possible effects on non-target species in vineyards should be accurately determined.

### Microbial resource management (endosymbionts) in insect vectors

A promising and sustainable option to limit the development and spreading of plant diseases can be provided by the understanding and the managing of microbiomes associated with insect vectors. This interesting strategy was proposed for the first time by Verstraete et al. (2007) and called Microbial Resource Management (MRM). It is known that all organisms are strongly influenced by the composition and interactions of their associated microbial community (i.e., cross signaling, cross feeding, aggregation, and adhesion) (Read et al., 2011). Numerous studies have described bacterial community, their dominant symbionts and parasites/pathogens to design a valuable microbial manipulation strategy against the targets, including microbiota associated with insect vectors potentially involved in the spreading of phytoplasma diseases (Marzorati et al., 2006; Douglas, 2007; Bressan et al., 2009; Gonella et al., 2011; Read et al., 2011; Fagen et al., 2012; Eljounaidi et al., 2016; Moussa et al., 2020).

One of the most interesting endosymbiont bacteria is *Wolbachia*, an intracellular symbiont found in nematodes, mites, crustaceans, and insects (Andersen et al., 2012). Being a reproductive parasite, *Wolbachia* can affect host fitness and its reproduction by causing cytoplasmic incompatibility, inducing parthenogenesis and feminization. *Wolbachia* showed to be able in modifying immune responses, protracting the latency period, and hindering pathogen transmission in insect-transmitted pathogens, like phytoplasmas (Chuche et al., 2017).

The obligate symbiont ‘*Candidatus* Sulcia muelleri’ (*Bacteroidetes*) was often found associated with Auchenorrhyncha, along with ‘*Candidatus* Purcelliella pentastirinorum’ and other *Bacteroidetes* symbionts (Moran et al., 2005; Gonella et al., 2011; Bennett et al., 2014; Moussa et al., 2020). These bacteria play an important role in providing essential amino acids to their insect hosts (Moran et al., 2005), opening novel and interesting avenues to study their utilization to manipulate the fitness of phytoplasma insect vectors. Further studies should be carried out to understand biological interactions between endosymbionts and their target organisms, and subsequently the efficacy of endosymbionts in open field conditions.

### Management of insect vector population and plant hosts of ‘*Candidatus* Phytoplasma solani’

The massive use of insecticides might be an efficient strategy for reducing diseases associated with phytoplasma, mainly vectored by monophagous insects living preferentially on grapevine (e.g., *Scaphoideus titanus* for Flavescence dorée) (Prazaru et al., 2023). Since *H. obsoletus* (and many other BN insect vectors) is a polyphagous species, whose life cycle is not restricted in the vineyard, but also involves mainly surrounding areas, hedges and forests, the use of insecticides cannot significantly reduce neither the vector population, nor BN incidence (Maixner, 2007; Mori et al., 2008); moreover, it can affect non-target organisms, with negative effects on biodiversity and natural ecology (Mori et al., 2014). Species distribution models investigated the influence of climatic and environmental factors on the occurrence of *H. obsoletus*, revealing that its incidence is associated with two main environmental factors: the amount of fine soil and the average annual precipitation. The application of these models can quantify the habitat requirements of *H. obsoletus*, representing a valuable tool for establishing the risk of high populations of the vector and optimizing its management strategies (Panassiti et al., 2013). In the vineyard, considering the attractiveness of suckers on *H. obsoletus*, the removal of the sprout along with chemical control before the adult appearance, can reduce the population density of the vector in the grapevine canopy (Picciau et al., 2010).

For the control of *H. obsoletus* on herbaceous plant hosts, chemical weeding, soil tilling and frequent cuts were proposed (Langer et al., 2004, Maixner, 2007, Mori et al., 2012); however, it should be considered that weed management may produce a relocation of insects, including those transmitting CaPsol, from wild species to grapevine obtaining a fast and significant raising of BN incidence in vineyards. Contrasting results were obtained evaluating the effectiveness of herbicides in different geographical areas. Kehrli and Delabays (2012) in Switzerland showed a low efficacy of glyphosate applications in autumn and spring on nettle roots in killing nymphs of *H. obsoletus*. The same experiments carried out in Italy and Germany significantly reduced the emerging adult vectors (Mori et al., 2014). However, environmental problems caused by the extensive use of chemical weeding, altering natural ecosystems, remains a concern. Weed management using herbicides may produce a relocation of insects from wild species to grapevine, including those transmitting phytoplasma, obtaining a fast and significant raising of BN incidence in vineyards . In addition, an important limit of chemical use is related to the low effectiveness achieved when reservoir sources grew in hard-to-reach areas surrounding vineyards (i.e., along ditches or hedgerows). In these cases, physical limitations or water contamination risks do not allow adequate weed control.

A remarkable control strategy was proposed by Mori et al. (2016) in vineyards located in Northern Italy, where nettle represents one of the most important inoculum sources of CaPsol. In this study, the reduction of nettle density and death of *H. obsoletus* nymphs was carried out within an integrated approach, including selective herbicide applications with frequent mechanical cuts of nettle.

Weed management should be carried out before the emergence of *H. obsoletus* from the roots of its host plants, followed by further cuts in mid-August and late summer, to avoid the relocations of adults inside the vineyard due to the lack of availability of their preferred plant species in the surroundings (Maixner, 2011; Mori et al., 2016). Alternatively, in organic vineyards, programmed cuts until late summer are highly needed to promote the development of perennial grasses, competitive with stinging nettle (Maixner et al., 2010).

To improve the effectiveness of BN control strategies, the identification of other weeds involved in the disease epidemiology plays a crucial role. Based on the feeding preferences and polyphagia of CaPsol insect vectors, it was possible to find new possible reservoir sources of phytoplasma strains, unveiling novel epidemiological patterns of BN. Considering *U. dioica, C. arvensis, A. vulgaris*, *V. agnus-castus* and *C. foetida* already included in the BN epidemiology, further studies have identified *Erigeron bonariensis* L.*, Helminthotheca echioides* (L.) Holub*, Sonchus oleraceus* L.*, Mentha arvensis* L.*, Datura stramonium* L.*, Potentilla reptans* L. and several other host plants involved in the BN epidemiology (Credi et al., 2006).

A multidisciplinary approach, including spatial distribution analyses (Spatial Analysis by Distance IndicEs) and molecular typing of CaPsol strains identified in the vineyard agroecosystem, showed that *Chenopodium album* L., *Malva sylvestris* L., *Polygonum aviculare* L., and *Trifolium repens* L. represented further putative inoculum sources of CaPsol in vineyards located in Franciacorta and Veneto region (Italy) (Mori et al., 2015; Quaglino et al., 2021). Moreover, a recent study demonstrated that plant species frequently employed for vineyard green manure (*Eruca sativa*, *Vicia sativa*, and *Polygonum fagopyrum*) can harbor CaPsol strains indistinguishable from those infecting grapevines. Based on this evidence, it is reasonable to suggest the exclusion of such plants from groundcover species selected for green manure (Moussa et al., 2023).

In light of several studies previously conducted, it was also determined that phylogeny of *stamp* gene-based molecular analyses allowed the grouping of bindweed-related CaPsol strains within the same phylogenetic cluster, showing that *stamp* gene can be the most suitable molecular marker for epidemiological studies on CaPsol-associated disease (Fabre et al., 2011; Cvrković et al., 2014; Murolo and Romanazzi, 2015; Kosovac et al., 2016; Pierro et al., 2018, Pierro et al., 2018a; Quaglino et al., 2019). Such studies reinforced the view that BN epidemiology is extremely complex and control strategies must be addressed to different broadleaves, depending on geographic areas and seasons.

### Utilization of push and pull strategy limiting *Hyalesthes obsoletus* populations in vineyards

Application of repellent (push) and trap (pull) plants to repulse or attract dangerous organisms in crops is an innovative and effective technology in sustainable agriculture. In Israel, *H. obsoletus* feeding choices were evaluated among 3 host plants (*V. vinifera, C. arvensis, V. agnus-castus*) by using a Y-olfactometer, revealing that *V. agnus-castus* was the most attractive host to the vector (Sharon et al., 2005). Although insects tested positive to CaPsol, the presence of the phytoplasma was never detected in *V. agnus-castus* plants; according to this, Sharon and colleagues (2005) suggested the use of chaste tree in the vineyard surroundings as a trap plant for *H. obsoletus* to prevent BN spreading in the vineyard. However, contrasting results were obtained in studies conducted in Montenegro vineyards, where CaPsol strains were identified in *V. agnus-castus* in addition to *U. dioica* and *C. arvensis* (Kosovac et al., 2016). Subsequent investigations have confirmed the role of chaste tree in the BN epidemiology in vineyards located in Northern Italy, not recommending *V. agnus-castus* as trap plant at vineyard borders (Moussa et al., 2019).

Considering this evidence , it could be possible to consider chaste tree as a plant trap only in specific geographic areas, such as Israel, where it can be included in the local management methodology. Up to now, in the European Continent such a pull strategy does not constitute a reliable approach.

## Conclusions

Phytoplasma are a wide group of plant pathogens causing considerable damages in hundreds of woody and herbaceous plants. Within grapevine yellows, BN represents a serious concern in many wine growing areas worldwide, where it has spread rapidly, impacting fruit quality and plant vitality.

The complex epidemiology of BN, involving several insect vector species transmitting CaPsol from multiple plant hosts to grapevine, makes very difficult the disease containment in vineyards. Traditional control strategies so far adopted have shown partial effectiveness with negative environmental impact, due to their generic range of actions, impacting natural ecosystems and its biodiversity.

In the last decades, several experimental trials have improved the sustainable control management of BN, targeting the main elements involved in its epidemiology and reducing environmental impact.

Considering the novel strategies and the effective results so far obtained, it is important to highlight that their efficiency is highly dependent on (i) cultivar and rootstock susceptibility, quality of propagating materials, (ii) application of strategies to increase the resistance of the plant or deter the feeding and transmission of the vector, (iii) the local environmental conditions, including weed composition inside and outside vineyards, insect vector populations, environmental parameters, habitat biodiversity, (iv) timing of weed management, and, (v) synergistic effects of multiple strategies adopted simultaneously and/or within the same plant growing season. Considering that, inside an integrated approach, we cannot intervene on the environment as well on the pathogen virulence, at least in an acceptable timely manner, the direct and indirect vector control, and the use of resistant grapevines remain the parameters to be preferably investigated for future BN control attempts. Further studies are needed to extend the promising results obtained in representative conditions to wider viticultural areas to validate the sustainable effective practices and avoid the conditions that risk increasing the inoculum load, the transmission and the appearance of BN symptoms, with heavy damage to the quality and quantity of the production. Interestingly, a recent study identified and characterized the functional activities of CaPsol effectors and pathogenicity factors in plants (Dermastia et al., 2023), opening new intriguing scenarios for new, additional management strategies based on interfering with such molecules.

## Author contributions

RP: Data curation, Formal analysis, Investigation, Methodology, Writing – original draft. AM: Formal analysis, Investigation, Methodology, Writing – review & editing. NM: Conceptualization, Formal analysis, Methodology, Resources, Writing – review & editing. CM: Investigation, Methodology, Validation, Writing – review & editing. FQ: Conceptualization, Project administration, Supervision, Writing – review & editing. GR: Conceptualization, Funding acquisition, Project administration, Supervision, Writing – review & editing.
